# An Enquiry Concerning That Disturbed State of the Vital Functions Usually Denominated Constitutional Irritation

**Published:** 1826-07

**Authors:** 


*An Inquiry concerning that Disturbed State of the Vital Functions usually denominated Constitutional Irritation.* By Benjamin Travers, F.R.S. Senior Surgeon to St. Thomas's Hospital, &c. &c. One vol. 8vo. pp. 556. Longmans, 1826.

The subject of irritation has excited great attention in this and foreign countries, during the last twenty years, as may be seen in the writings of Abernethy, Cooper, Hall and others on this side of the channel; and of a host of Broussaians on the Continent. And yet the investigation is in its infancy. It is one of the most important subjects that can occupy the attention of every class of medical practitioners; for the limits between irritation and inflammation are often so narrow—their features so much alike—their distinction so ambiguous—and yet their nature and treatment so different, that fatal mistakes are hourly committed by confounding the one condition with the other. On this account we were very glad to see a volume announced from the pen of Mr. Travers, on so interesting a topic; nor shall we fail, in two or three extensive articles, to give it every development which a journal is capable of affording.

§ 1. Mr. Travers properly introduces the subject of irritability, as a prelude to that of irritation. This principle in the animal economy has engaged many pens since the days of Haller. Irritability is not confined to any particular form of organization, as nerve, muscle, or blood-vessel, but is common to all, though in unequal degrees. It is not in the ratio of sensibility nor vascularity, nor muscularity—but, according to the importance of the organ in the functions of life. Thus, muscle retains it longer than any other texture, and the heart longest of all. Parts not essential to life, as the retina, or the muscles of a limb, may lose their irritability, and the patient live; but, if a vital organ lose its irritability, there is soon an end of life. As for irritability itself, we know nothing of its intimate nature —we only see it through the medium of the phenomena which it produces.

"Irritability is possessed by different individuals in various degrees, and susceptible in all, of infinite modifications. The tone of the nervous and vascular system, and the actions of the secreting and excreting organs, depending on the original frame and constitution, the degree of physical activity and mental energy, climate, diet, regimen, and in general, the nature and strength of the stimuli by which it is excited, are all modifying agents, direct or indirect, and lead to this variety." 7.

§ 2. *Morbid Irritability*. Every organ has its peculiar mode of irritability; and, so long as it neither falls short of, nor exceeds its due proportion, the harmony of the system is preserved. But various causes, internal and external, augment, diminish, or pervert the proportion, and then disorder or actual disease takes place. It is in this point of view that it becomes a particular object of attention to the medical practitioner, as exercising a vast influence over diseases and local injuries. An excess or deficiency of natural stimuli, or the operation of noxious agents, will convert healthy into morbid irritability—and a natural stimulus applied to a morbidly irritable organ, becomes itself an irritant.

"An irritable mind is one easily excited, and over excited in reference to the occasion, whether to joy or anger, fear or pity. An irritable stomach is nauseated by many ordinary articles of diet. An irritable bladder is continually parting with its secretion before the stimulus of distention can be supposed to act. An irritable heart, if quickened by exertion or strong mental excitement, becomes tremulous and palpitating. An irritable skin breaks into a rash from many slight causes of excitement, both of diet and temperature; a plaster, which is in others only rubefacient, acts upon it like a blister. Irritable fauces are slightly sore with a change of the wind, or exposure for a few seconds to a current of air; and an irritable retina is distressed to dimness by a full light, and, like the mind, disturbed by the surviving representation of transitory impressions." 9.

Irritability is either proper or sympathetic.

"First: as applied to the system. If the body is deranged through the medium of the mind, or, *vice versâ*, the mind through that of the body, it is sympathetic. But it is equally possible for the irritability of either to be singly and directly acted upon, and the other not affected, or, if at all, secondarily;—thus a man may lose his senses from a shock of mind, terror, and the body discover no sign of disorder but that which the loss of mental intelligence and expression occasions, the animal and vital functions going on as usual; as, to mention for one instance, the case of a young lady who was found playing with the fingers of a skeleton which had been placed in her bed, and who survived to old age in corporeal health, but without a glimmer of returning reason, in a lunatic asylum. Or, on the other hand, a limb may be wrenched off, and the man die in twelve hours, preserving his mind to the last. It must be admitted that the proper irritability of the mind is seldom exhibited, comparatively with the sympathetic; possibly in strict fact never, but we must be content to reason from the evidence of our senses." 10.

As applied to organs. If painful news take away the appetite—or pinching a tendon occasion sickness—if anxiety or apprehension excite the desire to pass urine or fæces—if the sight of savoury food excite the secretion of saliva—if certain articles of diet produce an eruption on the skin, or worms in the bowels cause temporary blindness—the irritability of the affected organ is sympathetic. In truth, the sympathies of health and disease are closely interwoven with this property; and the functions of life would be badly carried on without their cooperation.

Sympathies are considered by our author as associations founded on a reciprocity of sensations and actions—made up of sensibility and irritability. Some are direct, as between organs served by branches of the same nervous trunk—others circuitous, as between organs remote but maintaining an indirect communication through the medium of the brain.

An excess of physical irritability in health, is coupled, though not invariably, with a similar condition of the mind. In disease, the existence of either, speedily calls up the other, and too often with great injury to the individual. The following passage contains much truth, and matter for profound contemplation.

"Mr. Hunter defines an irritable habit to be 'an increased disposition to act without the power to act with;' in another passage he describes, irritability 'over-action to the strength of the parts.' This definition appears to me to be founded on correct observation. Extreme susceptibility and consequent over-activity are invariably coupled with, and most probably depending upon weak and insufficient powers of constraint and resistance. The same principle which renders a part over-irritable renders it over-active. The balance of the system, adjusted by the state of even health, is disturbed by the preponderance or deficiency of either of its active functions, as by the imperfection or disease of either of its organs. A weak organ or constitution is one easily disturbed and put out of order, because it is continually excited to greater activity than it has power to support, greater, therefore, than is consistent with the harmony of the system. But an action may be morbidly excessive or deficient, independently of organization, and this irregularity, although occasional in its commencement, may become habitual. A too irritable nervous or vascular function is, therefore, as marked a constitutional peculiarity, as irritable lungs or skin. In a physical, as in a moral sense, every individual has a weak part; and this observation would as often apply to the function, viewed abstractedly, as to the organ. Circulation, or respiration, or nutrition, in one or other of their many intricate processes, is below par in tone. The absorbent capillary function is below par in scrofulous habits; the arterial, in the leucophlegmatic; the venous, in those disposed to local congestions; the exhalent, in the dropsical; and the pulmonary, gastric, hepatic, and renal, are respectively the failing functions in persons who become eventually the subjects of asthma, gout, jaundice, and stone. Habits of life influence the body and its functions, as education the mind: but original structure, as well as that altered by disease, has a marked influence on function, and constitution is, to a certain extent, hereditary." 15.

It is easy to illustrate the influence of an irritable temperament upon the consequences of casual injury or disease. We say, "such a person would be a bad subject for a compound fracture," and the meaning of this expression is well known. In some individuals the constitution seems ignorant, as it were, of a local injury, even if severe—while in others, the whole system sympathises—the spirits are ruffled—the nights sleepless—the appetite is gone—the pulse quickened—the tongue whitened—and symptomatic fever established, from very trifling local causes. The following examples are in point.

"Two gentlemen, about the same time, were the subjects of the common accident termed, a broken shin. The injury was slight and of the same extent in both. One, accustomed to treat such casualties in his own person, simply defended the part lightly with a thin soap plaster, and without intermitting his habits of much daily exercise, both on horseback and on foot, suffered no further inconvenience. The other, making the same application, and using the limb with reserve, was, in the course of three days, compelled to assume the horizontal posture for nearly three weeks, suffering from a painful inflammation of the absorbents of the limb, and glands of the groin, and of the absorbent vessels encircling the loins, accompanied with a smart degree of fever. The bruise was, in this case, followed by an ill-conditioned sore, which, for many days, admitted of no other application than an emollient poultice." —17.

A knowledge of the constitution of the individual, is, in these cases, of great importance, and hence the conviction of this, on the part of the patient, is not a mere prejudice, but founded on truth.

"The unfavourable influence of an irritable frame of mind in the severer injuries and operations, is so universally admitted, as scarcely to require illustration. The fear of pain will often suspend the sense of it, as every one knows who has had occasion to lift the dentist's knocker. But it is the fear of death which operates with real and serious force against the best efforts of human skill, and this is excited in a degree, professionally speaking, by no means corresponding to the occasion. It is rare indeed for a person to be harrassed with this apprehension, who is approaching that awful crisis. It has been observed, that when the state of trembling alarm is not kept alive by attempts to appease it, the mind, ceasing from further conflict and struggle, gradually subsides into the more favorable condition of tranquil fortitude and resignation.

"The following are examples of mental influence.

"A lady, Mrs. S——, who, concurring, as a point of duty, with the advice of her surgeons, reluctantly submitted to the removal of a small tumor in her breast, unexpectedly, and without any apparent cause, died on the morning following the operation. It was then, for the first time, ascertained, that she had prognosticated her death, and the impression that she should not survive had taken so strong a possession of her mind, that her minutest household arrangements were preconcerted, as appeared by the papers found in her cabinet.

"The following fact, however extraordinary, comes well-authenticated to me, and was, as I am informed, much the topic of conversation at the time of its occurrence.

"About forty years ago, a young lady, afterwards Mrs. W. rallied her companion aloud for listening to the predictions of an itinerant gipsey, when the latter malignantly threatened her to beware of her first confinement. She was shortly afterwards married and became pregnant, and, as the period of her confinement approached, it became evident to her friends that the remembrance of the wizard malediction began to fasten upon her spirits. The circumstances of her labor were natural, but she survived only a few days. The medical attendants, who were men of eminence of the time, stated it as their opinion that mental prepossession alone could be admitted as the cause of her death; not one unfavorable circumstance having occurred to explain it.

"The following case I select, being within my own knowledge, from others of a similar import, of which I have been informed by professional friends.

"A young lady, happily married, impressed probably by some unexpectedly fatal occurrence in the circle of her friends, entertained, from the commencement of her pregnancy, a morbid fear of death in childbirth; which, although unwarranted by any indication of unhealthiness, became, from its continuance and increasing strength, a source of anxiety to one of her immediate and confidential relatives. She was attended by a skilful and experienced accoucheur, who was also her relation. He assured me that the labor was in all respects easy and safe, and that not a single unfavorable circumstance attended it. The child was still-born and imperfect. The mother died suddenly, in six hours after delivery. Every region of the body was examined with care by an eminent anatomist, and presented the appearance of health." 22.

Mr. Travers saw a man expire suddenly on the operating table, during the preliminary steps of a strangulated hernia, where the symptoms and state of parts presented no cause whatever for such a melancholy termination. A man of colour, of middle age, robust, and apparently in good health, was received into the London Hospital, with a moderate sized aneurism of the femoral artery. He readily assented to an operation proposed to him. He fainted, however, on entering the theatre. He took some wine, and the operation was commenced. The ligature was applied, but not drawn. It had been observed that no pulsation could be felt in the tumour during the operation, which was attributed to the preceding syncope. On examination, however, it was found that the man was quite dead, nor could any resuscitative means avail. On dissection, both sides of the heart were found empty, and the lungs gorged with blood. There was no cognizable disease in any part of the body.

Violent efforts to resist pain, or suppress the language of complaint, are sometimes fatal, and at all times injurious. Thus a man who had been bitten by a cat, and in whom some hydrophobic symptoms had become developed, made an extraordinary resolution to command his spasms, while the excision of the bitten part was going on. He died in three minutes by the effort.

Surgeons well know the danger of operating on persons in a state of what is called "*rude health,*" and the importance of preparatory treatment in such cases.* And although persons in an opposite state—*delicate health,* often do better, they are by no means favourable for local injuries or operations. Every one knows how much more fortunate are compound fractures and capital operations in the provincial, than in metropolitan hospitals. It is owing to the subjects of the former being principally agricultural labourers who have breathed pure air, and who, by constant and healthy exercise, have invigorated the processes by which the body derives its support and disposes of the surplus. Whereas, the artizans in the manufacturing districts of crowded cities are not only condemned to breathe a vitiated atmosphere, but are, for the most part, denied the advantages of good nourishment and proper exercise. But of all classes, the draymen, coal-heavers, and multifarious tribe of gin-drinkers, are the most unfavourable subjects for severe injuries, and sudden attacks of acute diseases.

"A pure stimulus, as that of alcohol, contains little nutriment, and having, when taken in habitual excess, a constant tendency to incapacitate the organs of digestion, impairs, and at length destroys, appetite. Malt liquor, when taken in such excess as to form the chief support of life, operates in the same way upon the digestive organs; but its stimulus being less potent, as well as its nutrient matter considerable, its effects upon the constitution are less obvious; under ordinary circumstances, perhaps less injurious. Yet no description of patients fare worse than brewers' servants, under the severe casualties to which they are exposed. They struggle with a morbid plethora. The debauchee of high life levels, in respect of constitutional strength, with the low drunkard of all denominations; and both require a mode of treatment, under severe injury, the very reverse of that which is adapted to the

* We agree with Mr. Travers in looking upon the state of "rude health," as a forced state—that in which the nutrient powers are tasked to the uttermost, and successfully struggle with a surplus of diet and stimulus, ridding the body of both by the action, at its full stretch, of every excreting organ. The subjects of this class are perpetually treading that precarious boundary which separates health from disease--and from which a very trifling shock or derangement of some important function may readily precipitate them into actual disease.

man, who, from his occupation and habits, labors, when disease overtakes him, under what may be termed a nutrient plethora; for while the latter will be infinitely benefited by full blood-letting and other means of reduction, wine and opium can alone save the former." 28.

Nothing can be more true than the sentiments contained in the foregoing extract, and it is really wonderful that men will not, for the sake of luxury, abandon the modes of fashionable life, and attend a little more to the dictates of Nature and reason.

In this place Mr. Travers passes a pretty severe satire on the "oracular decrees" of certain eminent men respecting dietetics, considering them as sickly fancies—"Ægri Somnia" which will no more satisfy the minds of unprejudiced persons than the prescribed rations will satisfy their stomachs.—It is, indeed, vain, if not improper, to think of confining each individual to four ounces of food three or four times a day, to be taken without drink. This quantity would be too much for some stomachs, and would not be nearly enough for others. We agree with our intelligent author that "every man's stomach informs him with as much accuracy as his moral sense distinguishes right and wrong, when it is indisposed for food—when it has received enough—and when it is overloaded—and his personal experience not only instructs him earlier and better than any one can inform him, what articles of diet to select and what to avoid, *but is the only process by which he can obtain the information correctly*." The last part of this sentence requires qualification. Many persons affected with indigestion, and feeling much debility, run into fault, with the greatest circumspection, by taking food of too rich a quality, which gives a temporary stimulus to the stomach and to the whole system, though it is ultimately the cause of much trouble in the digestive process, while the patient is unable to connect cause with effect. Here the advice of the physician will be of great consequence, and may save the individual many an uncomfortable sensation, and fit of gloomy despondency.

"A parliamentary enactment would be quite as reasonable as a tabular regulation, for the quality and quantity of aliment which people are to consume; and truly it were a sad omen for the nation, if the enervating refinement of the age had reduced our organs to this extremity of imbecility; for that, if such a conceit were generally acted upon, it would lead to this result, and make abstinence a merit of necessity, there can be little doubt. It is hardly possible to conceive a more hideous catalogue of evils than that which follows in the train of animal impoverishment; of the physical consequences we may form some idea, but it would be difficult to estimate the extent of its moral influence on mankind. Neither the physical nor the mental appetites and powers of the individual approach to a state of uniformity, but are, in the highest degree, variable. Man is the creature of circumstance; if he were not, he would be shorn of all his nobler faculties. Emulation, enthusiasm, and all the elevating impulses of his nature would be dormant, and the world would be sunk in a state of senseless apathy." 30.

But, however we may differ about the quantity and quality of our food, and the proper periods for taking it, all must agree respecting the invigorating efficacy of a pure atmosphere, both in preserving and restoring the functions of health. Whoever will take his daily station on any of the piers of Brighton, Ramsgate, or Margate, and watch the countenances of those invalids who fly from the smoke and turmoil and phrensy of London, to gulp a few mouthfuls of sea air, will be surprized at the effects produced by this short excursion. Every day gives the countenance a deeper shade of health, till in a few weeks, sometimes in a few days, we miss a whole train of individuals, who are attracted back to the pandemonium, again to suffer the original deterioration that drove them to the coast.

But to return from this digression respecting food and air. Mr. Travers, observes that certain natural states, as pregnancy and lactation, are unfavourable to surgical operations. And can we wonder at it. An important process is carrying on in the animal economy, the very disturbance of which can not but be hazardous—what then must be the consequence of both the disturbance of this process and the necessity for another new restorative action being set up? Mr. Travers operated for exomphalos on a lady, in the 5th month of pregnancy. The operation was performed early, and promised well, the function of the canal being restored. A diffused peritoneal inflammation, however, destroyed life.

"On the other hand, I was not long since called to operate for strangulated umbilical hernia on a young woman, fifty hours after child-birth, who had been laboring under symptoms of strangulation since the period of thirteen hours from her delivery. Although the protruded omentum and the gut were in part gangrenous, and a temporary artificial anus ensued, she recovered favorably and is now in perfect health. Had the operation been performed prior to the relief of the uterus, the issue, I feel confident, would have been fatal." 32.

From a middle-aged woman in full lactation, a scirrhous tumour was removed. The milk sprung as freely as blood under the knife. She died on the sixth day, of inflammation of the pleura. Organic disease of any of the important viscera, as of the lungs, liver, uterus, &c. is unfavorable to a surgical operation, as well as to any casual supervening acute disease. Inspection of the body, after death, frequently explains the cause of want of success in the best conducted operations.

The gradual reduction of the constitution which occurs during the progress of local disease, is, we all know, favorable to a capital operation, which may be necessary for the removal of that local affection. But this reduction may be carried too far, and the powers of life rendered unequal to the process of restoration. Very trifling operations, in a state of health, have proved fatal. We have seen two instances of crooked fingers being removed for mere convenience, the penalty being death. We lately saw an inconvenient toe removed from the foot of a most interesting young lady, the operation being performed by one of the first surgeons of this metropolis. She had a most narrow escape from tetanus. Mr. Travers has met with similar occurrences. The practice of the lately retired Mr. Geo. Young, of the city, may be deserving of record here.

"The following is an illustration of Mr. Young's practice in his own words. 'A healthy carman came under my care, with a loose cartilage in the right knee-joint. It had several times occasioned him to fall suddenly, and he was very anxious to submit to an operation to get rid of it. It appeared to me desirable to accustom him, before the operation, to the reduced diet, rest, and restraint, which would be necessary after it. He accordingly kept the house. On the second or third day of his confinement, I put on the roller and bound on the back splint, exactly as I intended to do after the operation, to keep the limb perfectly steady. This confinement of the limb occasioned a restless night, some fever, a whitish tongue, a quickened pulse, a little headache, spare and high coloured urine. He was very unwilling to continue the bandage and splint, to which he ascribed (and justly) all his constitutional disturbance, and the utility of which, prior to the operation, he could not at all comprehend. This circumstance, however, forcibly suggested to me the importance of accustoming him to restraint; it was therefore continued; the excitement which it had produced gradually subsided, and when I found that the bandage no longer occasioned any irritation, I performed the operation. Not one untoward symptom arose, the constitution was not in the least ruffled, and the wound healed by the first intention." 38.

### CHAP. II. EFFECTS OF LOCAL INJURY ON THE CONSTITUTION.

Irritation is, as was before observed, local or general. The phenomena of this state are chiefly displayed through the medium of the nervous system—and is thus distinguished pathologically from inflammation, which appertains more to the vascular system. The relation of the two conditions, however, is as intimate as that of the two systems themselves. Inflammation has been sometimes considered a healthy, because a healing action. In the same sense, the minor degrees of irritation frequently have a salutary purpose, and conduce to the preservation of the system. Mr. Travers objects to the term "irritative fever," as synonymous with irritation, "because irritation and fever are, in their nature, as distinct as irritation and inflammation," although their reciprocal affinities, are as intricate and complicated as those of the nervous and vascular systems. This is a true remark. We shall see a person of a nervous or irritable constitution, become, from some mental or corporeal disorder, affected with white tongue, quick pulse, restless nights, loss of appetite, torpid or vitiated secretions—in short, with many of the phenomena of fever. But, upon an attentive examination, we shall find some essential characters of pyrexia absent. Thus, although the tongue may be as white as paper, there may be little or no thirst—and although the pulse may be very quick, there will not be a corresponding increase of temperature on the surface, and so on. Still it is difficult, in words, to lay down the line of demarcation between some states of constitutional irritation and fever, though the experienced practitioner will generally distinguish them in practice.

*First.* Local irritation then, is demonstrated best, by an alteration in the habitual and proper sensation or action of a part—as a depravation or suspension of function in an organ of sense—an aberration or delusion of perception—a vitiation, suspension, or redundancy of secretions—an irregular and involuntary action of muscles, or a partial paralysis, &c.

*Secondly.* By pain, unattended by any other sign of inflammation.

"The irritable joint, breast, testicle and prostate gland, give no evidence of inflammation. The irritable organ or its vicinity, has in many instances been affected by sub-acute inflammation at some former period*, but not such as to leave any trace of an altered structure. A carious tooth sometimes occasions a tic douloureux of the dental nerve. Worms in the maxillary sinus have given rise to the same painful affection of the sub-orbitar nerve. Some calculous concretions, which form

"* The most obstinate case of irritable breast which has fallen under my notice, was supposed to originate from a needle having formerly penetrated the integument of that gland. In another female a similar affection of the knee-joint was attributed to the same accident. After the lapse of many months from the extraction of the needles, and in the total absence of inflammation, the complaint continued in either case unrelieved and immoveable."

in the kidney, create severe local pain in those organs without a symptom of inflammation. The remedies for inflammation afford no relief, but gentle exercise and medicines which act chemically upon the concretion, remove it. The urinary organs, rectum, and uterus are, as much as any parts, subject to pain unattended by inflammation. But of all parts, the membranes of the brain and stomach probably suffer most in this way. A blow on the stomach, a compression of the testicle, colic from indigestible matters in the intestines, and the paroxysms of stone in the gall and urinary bladders, are frequent and familiar examples of the insufficiency of transient local irritation, though accompanied by acute pain, to excite inflammation. When irritation is unaccompanied by inflammation, it may be, and often is, remote from the seat of the pain and other signs of disorder by which its existence is known. The disease called tic douloureux exemplifies this fact abundantly. Numberless modifications of pain—viz. that degree of uneasiness which attracts attention to a part, and seems to admit only of a negative description, the varieties of prurigo, sub-acute and chronic inflammation about the orifices of canals, producing elongations and excrescences, or exulcerations and fissures, and the spasmodic contractions of the sphincters—are for the most part demonstrative of a remote local irritation unattended by inflammation." 42.

*Thirdly.* Irritation, when acute and permanent passes into inflammation, proportioned in degree to the severity of the irritation, and to the habit of the patient. Local irritation also, when in a high degree, becomes transferred to the constitution, either with or without inflammation—as in severe cases of disorganization, chemical or mechanical—extensive burns—crushed joints—compound dislocations, fractures, &c.

The terminations of local irritation are in resolution—local inflammation—or constitutional irritation. This last subject it is now our author's business to investigate.

2. *Constitutional Irritation.* When this arises from injury or inflammation, and changes from local to general, it becomes imminently hazardous—inasmuch as disorder of the whole is of graver importance than disorder of a part. The causes, especially in a surgical point of view, are the various kinds of injury, as compression, concussion, lesion, or disorganization, whether chemical or mechanical—inflammation, the result of local injury.

" Constitutional irritation I consider to be of two kinds, direct and reflected; by which arbitrary distinction I mean to imply, that the first is wholly and immediately derived from the part, commences and is identified with the local mischief, and the constitution has no share in its production. The second, on the contrary, originates in a peculiar morbid state of the constitution to which the injury or inflammation has given birth, or, it may be, previously existing. The first is truly symptomatic, never originating spontaneously, and being immediately induced by the local irritation, is capable of being essentially mitigated or arrested by its removal. The second is occasionally purely idiopathic, and being oftener the cause than the effect of the local action, is seldom influenced by local treatment. In the first, the local appearances are conditions depending on local causes—in the second they depend on constitutional causes. The symptoms characterising direct constitutional irritation, are, in the nervous system, rigor, delirium, convulsion, coma—in the vascular, the fever of phlegmonous, suppurative, ulcerative, and gangrenous inflammation. Those which belong to reflected constitutional irritation, are, in the nervous system, epilepsy, tetanus in all its modifications, and other anomalous forms of spasm, mania, &c.—in the vascular system, the fever accompanying scrofulous and carcinomatous inflammation, erysipelas, carbuncle, &c. I deem it no objection to a division of this sort, that the parts are so blended and interwoven as to render the outline here and there obscure or even imperceptible;—it is a circumstance unavoidably resulting from the nature of the subject." 49.

Our author next proceeds to consider the influence of contingent circumstances in the production of constitutional irritation. These are—1mo. The texture or organ injured—2ndo. The kind of injury—3tio. The magnitude of the injury—4to. The subject of the injury.

1. *Texture.* The natural and healthy sympathy of the constitution with a local injury is in proportion to the sensibility of texture of the injured part. Thus, lesion of tendon, ligament, or cartilage, per se, will excite less constitutional disturbance than that of skin, muscle, or mucous membrane. But injuries are generally complicated—and, at all events, the sympathy with the injury, at the moment of its infliction, is by no means the measure of the constitutional irritation which is, sooner or later, to follow.*

In the following passage it is evident that our author is somewhat in contradiction with a previous statement. "The fact is, with few exceptions, that injuries of parts of minor sensibility and low organization, induce the highest and most alarming degree of constitutional irritation." The power of reparation in parts being according to their endowment, the system is unusually excited to set up and sustain the restorative effort in injuries and morbid affections suddenly induced, of more dense and inert textures.

* The inflammation excited by the injection of the tunica vaginalis for the radical cure of hydrocele is invariably, in my experience, most languid in those persons who suffer most severely from the operation." 51.

"The mucous membrane, which lines the fauces, alimentary canal, and urinary organs, and the serous, immediately investing the viscera of the head, chest, and abdomen, arouse, when the subject of injury or inflammation, the highest degree of constitutional sympathy, from their intimate relation to the vital functions. But, if it were possible to abstract the weight of this consideration, I should say, that the constitutional irritation is more intractable in cases of injury followed by acute inflammation of the tendinous sheaths and ligamentous capsules; or of the fibrous membrane, which forms a close covering to the bones. At least, they differ thus essentially in character. The constitutional excitement characterising the former, assumes the more intelligible and bold form of inflammatory fever, capable of being combated by a vigorous reduction of the animal powers; while that of the latter presents the complicated phenomena of nervous derangement, commingling with, and mysteriously modifying, if not annihilating, the sympathetic fever." —52.

Our author makes some short, but judicious observations on the lesions of integument, muscle, tendon, bone, blood-vessels, absorbents, nerves, and internal organs, for which we refer to pages 52—56, and then comes to the *kind* of lesion. From this part we shall introduce an extract in the author's own words.

"I believe the danger attending punctured wounds depends upon the following circumstances. First. Penetration of some fibrous membrane; for if they do not penetrate deeper than the cellular substance, they are unimportant, even though suppuration ensues, because a cellular abscess quickly develops itself, and readily discharges, or is relieved of its contents. Secondly. The propensity of fibrous and ligamentous membranes to continuous inflammation. Thirdly. The form of wound being unfavourable to the discharge of fluids secreted under inflammation. Fourthly. The occasional puncture of a nervous filament, the division of which would be unimportant.

"An inflamed fascia betrays, for a time, no symptom of its existence but that of local tenderness. Where matter is formed, an erysipelatous blush covers the surface and a uniform diffused swelling takes place, though, for a long time, without fluctuation. The symptoms following a pricked nerve are developed in the course of the nerve, or in the system to which it belongs, more than in the wound. It is not, therefore, the form of wound in itself considered, but the form co-operating with the depth and the textures implicated, the obscurity attending suppuration in its commencement and progress, and the consequent neglect of repose and precaution, which render punctured wounds serious in their consequences.

"Contused and lacerated wounds seldom admit of adhesive inflammation: being the result of violent læsion, the texture is in part disorganized, and sloughing ensues. The tone of the surrounding parts is destroyed. If the dressing be such as to act like a stricture or ligature, from the tightness of its application, or the swelling which is occasioned by re-action, evil consequences ensue. If the injury be confined to cutaneous texture, erysipelas ensues, as we see in the scalp—if it extend to muscular or tendinous fibre, as in lacerations of the hands and feet, tetanus is the consequence most to be apprehended. It is owing to the widely dissevering and destructive nature of the injury, and the processes of inflammation which must ensue, and not unfrequently, to their interruption and exasperation by ill adapted treatment, that the system sympathizes so deeply in this form of wound. Gunshot wounds come under the last denomination, with the additional irritation created by foreign substances, and are frequently productive of tetanus. Comminuted fractures are accompanied by laceration, contusion, or irritation of muscles by spiculæ, and are, for this reason, especially dangerous."—60.

The extent of an injury must, of course, have considerable influence on the quantum of irritation. The exposure of the entire muscle of the calf of the leg—or half the scalp—or balls performing the circuit of the trunk—extensive burns, &c. are calculated to excite our fears for the constitution; though it must be admitted, on the other hand, that mischiefs of much smaller dimensions, as slight abrasions, punctures, and the like, are now and then fatal in their consequences. The magnitude of an injury is felt in two ways—in its immediate, and in its secondary effects. Either the system breaks down at once, and the vital functions are stagnated by the appalling shock which they have received; or, having withstood this, the constitution, with diminished powers, engages in the unequal conflict—erysipelatous or gangrenous inflammation is set up, and it sinks exhausted under the wasting processes which must clear the way for reparation.

In respect to the *subject* of the injury, it is evident that age, sex, and condition—climate, regimen, and other adventitious circumstances, must modify the moral and physical constitution of the patient, and thereby influence the result of injuries.

Scrofula in the system will often mask the symptoms of constitutional irritation, or cause them to appear comparatively lenient at first, so as scarcely to disturb the general health, and the onus of the mischief falls, as by accumulation, upon the more advanced stages of its progress. Reflected irritation is first displayed in the formation of the hectic paroxysm, and ultimately in the signs of consentaneous morbid changes in internal organs.

### CHAP. III.—DIRECT IRRITATION.

The following are the more frequent causes of this condition. 1mo. Sudden, extreme, and unremitting pain, and certain affections of the mind co-operating with bodily disease. 2ndo. Injuries and operations. 3tio. Inflammation, the result of injury, or operation, terminating in suppuration or in gangrene. 4to. Exhaustion from hæmorrhage or colliquative suppuration. 5to. Poisons, animal, vegetable, and mineral. Mr. Travers now proceeds to offer examples in illustration.

§ 1. *Bodily Pain—Mental Impression.* Pain itself, in a certain degree of intensity and duration, has been often fatal. Difficult and protracted parturition has offered some melancholy examples of this.

"There is a case in which, with an unconfined state of bowels, abdominal after-pain, aggravated by pressure, augments, at no distant period from delivery, to a degree sufficient to induce the belief that puerperal inflammation exists: the pulse is accelerated, and notwithstanding its want of power, and a general expression of feebleness, the practitioner, suspicious of the pain, takes away a full quantity of blood. No satisfactory result is obtained; the pulse and the patient sink together, and a fatal coma succeeds. This is a pain not of inflammation but of irritation, and would have a better chance of relief from laudanum than the lancet." 67.

Our readers are aware that Dr. Hall has drawn the attention of the profession very strongly to this important class of affections. A case is introduced by our author, in illustration, which was communicated to Dr. Merriman by his brother-in-law, Dr. Gooch. We shall abbreviate it.

A lady, aged 31, fell in labour of her sixth child, and on examination, a large fleshy substance was found presenting. The membranes protruded and were ruptured; but the accoucheur was obliged to turn and deliver by the feet. The placenta was expelled, and every thing appearing safe, he left the patient at seven in the morning. At three he was sent for, the lady having such violent pains that she thought there must be another child. The state of the abdomen negatived this idea, and an opiate was given. On returning at eight in the evening, he found that the pains had been as violent as ever. A soft tumour was felt pressing on the os externum. There was no hæmorrhage. An anodyne mixture was given, but the pains continued, with violent expulsive efforts all night. Next morning the patient had a languid pulse and a pallid countenance. A large fleshy tumour had been forced out of the vagina. She continued to suffer and to sink through the rest of the day, and expired in the evening.

On opening the body the uterus was found contracted, but its mouth was dragged down as low as the os externum, by a tumour which grew from it by a broad base. It was of a livid colour, and weighed nearly four pounds.

This case is curious, as presenting the rare occurrence of polypus in pregnancy, and as a striking proof that pain can destroy life.

Mr. Travers thinks that, in some mortal injuries, as rupture of the stomach, gall-bladder, urinary bladder, &c. death is often produced by pain, before the consequent morbid changes are so far established as to make it credible that the result is to be ascribed to their influence.

"I was called some years ago to a gentleman writhing with pain in the abdomen, which he had endured for the space of two hours previous to my seeing him, and described as unlike any that he had ever experienced. He accosted me in these words: "Doctor, if you cannot put an end to this pain it will very soon put an end to me." So true was his prediction, that in twelve hours from a state of comparative health (he had been at the theatre the preceding evening) he was no more. His disease was an ulcer in the pyloric portion of the stomach, which had perforated its coats, and allowed of the escape of its contents into the general cavity." 72.

Our readers well remember a nearly similar case which we stated lately, that came under our care with Mr. Stanley and Mr. Best, in the person of a gentleman in the neighbourhood of Covent Garden.

Poisons taken into the stomach seem to destroy life through the medium of the nervous system, as characterized by overwhelming pain, without competent organic change to account for the extinction of vitality. In all cases, Mr. T. observes, pain has its seat in the brain, being only a mode of sensation; but it is probable that the *expression of suffering* is often a fallacious criterion of the *measure of pain* endured, owing to the difference of temperament in individuals. Every texture has its characteristic sensation under irritation—and the different forms of injury and of inflammation have also theirs. There is a pain of the nerves—of the muscles—of the serous membranes—a pain of laceration, of division, and of distention, of suppuration, ulceration, &c. and the figurative terms, burning, pricking, shooting, lancinating, throbbing, &c. are in constant use to express these.

"In most instances of death from violent disorganization of texture, little, if any, pain is apparently endured. The shock suspends the sensibility of the system without deranging the mental faculties, although their vigor may be considerably abated. But where the symptom of excruciating and enduring pain is present, unaccompanied by the shock of violent injury, it excites and absorbs the faculties of the mind; renders the sufferer wholly indifferent to external objects, and insusceptible of domestic sympathy and the tender emotions; makes death an object not of terror, but of earnest and unceasing solicitude; and terminates life by exhaustion in a very few hours." 76.

In operations, protracted by unforeseen difficulties, as in lithotomy, the patient has begun to die upon the table. The same happens in protracted parturitions. But where pain, however severe, has intermissions, as in tic douloureux, it can be borne for a long time, though even so, it gradually undermines the constitution, as was proved in the melancholy case of the late Dr. Pemberton, whose remarkably athletic and robust frame became emaciated to a shadow by mere corporeal suffering. "Pain," he was wont to say, "is the greatest sedative in nature."

"The first effect of intense and unremitting pain is precipitation of the action of the vacular system, with corresponding sensorial excitement; but neither of these signs are of long duration. The pulse, which has at first a strong bound or jerk, soon becomes small, tremulous, and irregular, or fluttering; the countenance, the features of which in the first instance are braced and compressed by a strong convulsive expression, quickly becomes relaxed, hollow, and ghastly. The extreme preternatural mobility of the muscular system, indicative of great restlessness, disappears, and a state of stupefaction and indifference to surrounding objects announces the state of exhaustion. If pain be the result of inflammation, its gradual increase prolongs the stage of excitement. If on the other hand its accession in an extreme degree is instantaneous, as from breach of texture or the operation of any destructive agent upon the system, the stage of excitement is comparatively short-lived. And when the description and extent of mischief inflicted are such and so aggravated as to produce a sensorial paralysis, evinced by partial stupefaction without absolute loss of consciousness, it so far neutralises or renders void the effect of painful impressions, as to admit of a direct prostration of the system without reaction. A large loss of blood at the moment of injury tends invariably to this result; that is, it cuts off the stage of excitement." 78.

The effects of mental perturbation, as co-operating with bodily disease, have been already alluded to, and more examples, in illustration, are here introduced by Mr. Travers. We shall glance at one or two. An elderly and somewhat corpulent lady was, with difficulty, prevailed upon to have a tumour removed from her breast. There was nothing, however, in the operation to create alarm in the friends or the medical attendants. Yet it was succeeded by continued and unvaried prostration of strength and spirits, with a quick and very indistinct pulse, restlessness, and an extreme irritability of the stomach, which rejected both food and medicine. Low delirium and stupor came on, and she soon died. The lips of the wound were slightly adherent, but on separating them, its cavity was filled with sanies. The following graphic sketch is closely copied from Nature.

"Every medical man of observation admits and appreciates the influence of the mind in disease, and how much his prognosis is influenced by this consideration. The variety evinced in the dispositions of different individuals in similar circumstances is remarkable. Some patients are mindful of the smallest attention, and grateful for it; obedient, hopeful; always looking forward to recovery, long and cheerless as is the journey; ever lightening the burthen to themselves and those about them, by a blessed spirit of contentment. Others, on the contrary, lie ruminating on the mischance; sullenly calculating the cost, if all proceed well; cast down by every adverse circumstance, and always anticipating worse; ever slow to acknowledge improvement, and selfishly regardless alike of the feelings they excite and the attention they receive. These though not imaginary, I grant, are extreme cases; there are however many intermediate shades. Such differences as these are not wholly to be attributed to moral causes, neither are their effects limited to the moral constitution; they have, on the contrary, a marked influence on the functions of life and the powers of recovery." 81.

Two interesting instances of the influence of previous insanity are adduced by our author, of which we shall sketch the particulars.

A man jumped, in an affray, from a window eight feet high, and sprained both ancles severely. Next morning he was brought to the hospital suffering much from the accident. Bowels freely opened, and an evaporating lotion applied. He was a little better. But in the middle of the ensuing night he was seized with vehement delirium, attended with quick hard pulse. He was freely bled and purged, and became tranquil. In the evening the pulse rose to 140, with fierce delirium. Temporal arteriotomy with a large blister—mercurial and saline purgatives, &c. The delirium continued with unabated violence, and on the following evening he died, 72 hours after the accident. It was ascertained, upon inquiry, that this man had been the subject of temporary insanity, and had been more than once confined in a lunatic asylum. On inspection of the body, a slight serous effusion was observed between the dura mater and tunica arachnoidea, but no other morbid appearance in either of the visceral cavities, except the chronic change so often seen in the liver of a spirit drinker.

The following case was communicated by Mr. Soden, a gentleman in extensive practice in Bath.

"A young man, nineteen years of age, who had formerly been maniacal and confined in a lunatic asylum, was admitted into our infirmary with stone in the bladder. I operated upon him. The calculus was large, weighing eight ounces, and consequently required a free incision, but the extraction was effected with much less difficulty or violence than the magnitude of the stone led me to expect. Shortly after the patient was in bed he became very animated, and said he should soon be well now that the stone was removed. The animation increased, so that in two hours after the operation he could with difficulty be prevented from getting up. In five or six hours he was in such a state of mania that it was necessary to use the strait waistcoat. The maniacal symptoms continued till death took place, about forty-five hours after the performance of the operation. On opening the body I found the bladder thickened, but all the parts connected with the operation free from inflammation and in a favorable state. The brain could not be examined. In this case it is probable that the man's apprehension about the operation, the operation itself, and his joy at its conclusion, acted upon an organ which was predisposed to disease, and were the exciting causes of the fatal paroxysm." 85.

In the next section, our author introduces some cases of severe burns, as exemplifying the symptoms of direct irritation in their most aggravated forms. These kinds of cases are so familiar that we need not stop to detail any of them, but pass on to the remarks which our author makes on them.

He thinks it may be inferred, from the history of severe burns, that the first three days include a period of imminent danger. When this period is passed, reaction may be considered as established, and, generally speaking, the injury has nothing more of a peculiar character. Infants, however, are sometimes suddenly attacked and carried off in convulsions, as late as the fifth day after re-action is fully established. This is a rare case. Aged or infirm persons may fall victims to sphacelus a week or ten days subsequent to the injury.

"It is remarkable to how late a period the faculties of the mind are preserved, although somewhat benumbed, so that to rouse them a strong external impression is often required. A patient in a case of fatal burn, after the first expressions of anguish have subsided, nearly resembles a person stunned by a fall, or as much as possible stupefied with liquor, without suffering an actual suspension of his senses. Inspection demonstrates fullness of the veins of the brain and its membranes, and effusion beneath the arachnoid membrane, confirming the symptoms which occur in the latter stage. But these appearances are too slight, and too frequently seen in the bodies of persons who die rapidly, to be much dwelt upon. The proximate cause of death appears to me to be a species of concussion, functional, not organic, by which the brain is deprived of its influence over the organ of circulation; for the symptoms of cerebral disorder are first manifested; secondly, a diminution of the power of the heart; thirdly, the respiratory function becomes impeded, as a necessary consequence of the two first." 108.

EXAMPLES OF FRACTURES, CONTUSIONS, &c.

*Case* 1. In the evening of July 2, 1819, our author was called to a fine lad of 13 years of age, who had received the charge of a musket (slugs) in his thigh. The charge entered about two inches from the trochanter major, and passed obliquely across the limb. The comminution of the bone was evident—no external hæmorrhage—the pulse could not be felt—countenance pallid—surface cold—pupils fully dilated. Yet he was perfectly rational when roused, but strongly disposed to stupor. Made no complaint of pain, but was troubled with insatiable thirst. He died without any alteration in the course of nine hours. On examination, it was found that the hip-joint was uninjured. The trochanter and upper part of the femur were shattered to fragments, and the surrounding muscles extensively lacerated, the artery being torn across. The effusion of blood was inconsiderable. The contents of the head, chest, and abdomen were perfectly natural.

*Case* 2. The wheel of a waggon went over the left leg and right thigh of the waggoner, at a village near London. Both bones of the left leg were fractured a little above the ancle joint, and the femur was broken transversely below the trochanter. The pulse was scarcely perceptible, and soon failed altogether—extremities were cold—lay in a state of stupor except when roused. He died within eight hours from the accident.

*Case* 3. From Mr. Green.

"A man fell from the roof of a coach, and in the fall suffered a compound fracture of the leg. He was replaced on the coach, and brought to London, a distance of forty miles. On his arrival at the Hospital, he appeared as if intoxicated, but recovered from this state in the course of a few hours. On the third day, without any considerable previous inflammation, the leg began to assume a gangrenous appearance. On the fourth day he became insensible; his breathing stertorous, and the pupils of his eyes dilated. The symptoms so much resembled apoplexy, that it was supposed some effusion of blood had actually taken place in the brain. The same night, he died, and on examination no morbid appearance whatever was found in the brain or other viscera," 112.

*Case* 4. We are tempted to give the particulars of one more case of this description of injuries. It was communicated to our author by Mr. Soden of Bath.

A young girl, 18 years of age, fractured her leg by jumping off a wall, at 1 o'clock in the day. The fracture was simple, and reduced by Mr. Hill, an hour after the accident. She was conveyed home (a distance of three miles) in a boat—making no complaint, and her pulse only seventy-two. At midnight she became restless, and kept tossing her arms and legs about —her face was flushed, skin hot, and breathing laborious. In the morning she was found in a state of coma, with quick feeble pulse, stertorous breathing, contracted pupils, cold skin, &c. The parts about the fracture were slightly swelled and bruised, but presented no appearance which could at all explain the formidable symptoms that had taken place. No injury could be detected about the head. She expired 31 hours after the accident. The vessels of the brain were turgid with blood, but no extravasation or other morbid appearance could be discovered.

The foregoing cases are quite sufficient to illustrate the dreadful effects which sometimes follow accidents, and we shall now introduce one or two instances of similar direct irritation succeeding operations.

*Case* 1. A stout lad, 15 years of age, had his wrist and hand literally smashed to pieces by a carding machine. There was little or no hæmorrhage, and he bore the amputation without complaint. He passed a quiet night, and at one o'clock next day had three copious green evacuations, after which he complained of nausea, rejected all diluents, and had a very quick and not very distinct pulse. Complains only of thirst and sickness. In the evening all the bad symptoms were exasperated. On the third day, no favourable change—extreme anxiety—cold clammy surface—livid countenance—purple extremities—laborious respiration. Wine and brandy were given, but he sunk, forty-eight hours after the operation.

The body was examined with the greatest care, but no morbid appearance could be discovered any where, except a slight erythematous blush on the villous coat of the stomach—an effect most probably of the vomiting which had occurred before death.

*Case* 2. (Communicated by Mr. Brodie) A patrole, aged 50, was admitted into St. George's Hospital, at 1 o'clock on the morning of February 5th, having received the contents of one of his own pistols in the upper part of the thigh. There was an extensive lacerated wound of the skin and muscles where the slugs had passed out. The trunk of the femoral artery escaped. He complained of little pain at first, but in half an hour it became excruciating—pulse regular, full, and strong, at 88. Nothing but amputation at the hip-joint presented any chance of salvation, and Mr. Brodie immediately proceeded to the operation. The amputation was accomplished without much difficulty, the artery having been previously secured under the crural arch. After the operation the patient was very faint, and the pulse 144, weak and irregular. He became sick and vomited, but complained very little of pain in the stump. The stomach would not retain any thing. The pulse got up to 161. An enema with 40 drops of laudanum was prescribed. Low delirium came on, and he was carried off in a convulsion 20 hours after the operation.

*Remarks.* The predominant symptom in which these cases coincided, is characteristic of the most intense degree of shock, viz. the early and unappeasable irritability of the stomach.

### OPERATIONS FOR CHRONIC DISEASE.

Mr. Travers confines his observations, under this head, to some remarkable cases of lithotomy in children, proving fatal by direct irritation. We shall, as usual, condense some of the most remarkable instances, for the benefit of those who cannot have access to the original work.

*Case* 1. A child three years of age, was operated on for stone in St. Thomas' Hospital. The operation was admirably performed, and did not exceed *one minute* by the watch. The little patient had a slight shiver after being put to bed, and the temperature of the body did not recover. He was inclined to doze—became slightly convulsed, and died at 2 o'clock the following morning.

The same day a boy, five years of age, was operated on by the same gentleman, and though the operation lasted longer, the little patient recovered without a bad symptom.

*Case* 2, 3, 4. A child aged 3 years, underwent the operation under favourable circumstances. In an hour he was chilled—

a stupor came on, and he gradually sunk, and died the same night. About the same time a lad of sixteen years was operated on in the same hospital—and the symptoms above described came on and carried off the youth on the same evening.

In none of these cases was the calculus very large, nor had hæmorrhage to any amount succeeded the operation. So many unfavourable results, in a short time, led to minute inquiries respecting the previous habits and diet of the patients—and the conclusions formed from the information thus obtained, led to the treatment so successfully adopted in the following case.

*Case* 5. "A child, six years old, became restless within an hour after the operation, which had been in all respects favorable. Between five and six o'clock, the operation having been done at noon, he was cold, faint, without a pulse at the wrist, and apparently dying. In this state, gin and æther diluted with barley water were got into the stomach, and repeated at intervals, until the pulse acquired a steady beat, and the surface its natural warmth. Its effect was immediate, and as salutary as could be desired. In diminished quantities the cordial was occasionally administered during his convalescence, which was from this time uninterrupted." 137.

It was, in fact, ascertained that the parents of these children had been in the habit of giving them gin to allay the severity of the paroxysm in micturition. The same treatment has been since resorted to in cases where similar symptoms had presented themselves. The following case illustrative of this principle was communicated to Mr. Young, and as it is short, we shall give it in his own words.

" 'A livery-stable keeper in Moor Fields fell with his horse and broke his leg; I saw him soon after the accident; yet he was calm and self-possessed, and took a part in directing those who removed him to his bed, and who afterwards assisted me in placing his limb properly. On the following morning, I was informed that he had taken little or no notice of any thing; that he had refused whatever was offered to him, and that he had not moved, but lain as if in sleep, as I then saw him; breathing very gently, with a pale, cold surface; a thready and rather quick pulse. I could rouse him by speaking firmly; he answered rationally, put out his tongue, said he had no sort of pain in the limb, which was cool, and not in the least swollen. I urged the necessity of his taking nourishment, and, as he preferred porter, I gave him by spoonfuls, a pint, and directed that he should take beer caudle freely. He gradually recovered, but several days passed before he felt quite restored, or complained of pain, or that any swelling was perceivable in the broken leg.

" 'Precisely the same train of symptoms, ending however in the gradual extinction of life, followed the removal of a large fatty tumor from the fore part of the thigh of a middle-aged woman. The operation was quickly over, and had been borne with exemplary self-possession." ' 139.

In the preceding and other cases, the youth of the patients, the debility induced by previous suffering, the influence of terror or of pain, the inevitable loss of blood, and, in some, the privation of an habitual stimulus, when really needed, may have been one or all accessory to these untoward results. When a degree of vacancy and stupor comes over a child shortly after being replaced in bed, and the countenance and general surface assume the paleness and coldness of death—the pulse being small, rapid, and indistinct, the patient becomes, in a few hours, comatose, and in that condition sinks. This is the extreme state of prostration from shock. There is a close analogy of symptoms between this condition of children and of the subjects of severe burns and complicated injuries. In two of the cases related by Mr. Travers, the patients died in convulsions. The unavoidable effusion of blood in operations, though insufficient to create alarm for the patient's safety on that score, obviously predisposes to the convulsions of children.

"The phenomenon of convulsions is invariably coupled with the state of cerebral irritation. This irritation may have been propagated to the brain from any suffering organ, as from a wounded muscle or nerve; from worms, or sordes in the primæ viæ, suppressed catamenia, or tension of the gums in dentition; or it may arise from the pressure of bony spiculæ on the brain; lesions, vascular congestion, or effusion in that organ. In either case, convulsions are symptomatic of disturbance, amounting to an interruption or temporary suspension of the cerebral influence, whatever that may be.

"We see these spasms arise in apparently opposite states of the system—in the plethoric and the ex-sanguine—in the robust and the debilitated—in congestion and in effusion—in acute inflammation and in destructive ulceration.

"Severe local irritation will occasion vascular congestion, and ultimately effusion in the brain, and hence gives rise to convulsions.

"Inflammation, being a frequent cause of local irritation, also operates to produce convulsions. When the irritating cause is in its nature such as to admit of removal, the convulsions cease upon its removal. Thus I have seen them cease after removing a spiculum of bone by the operation of the trephine; also after the discharge of confined matter; by blood-letting in parturient women; by the same and the operation of purgatives in recent acute inflammation, as in hydrocephalus, prior to effusion. The tendency of a local irritant to produce convulsions is augmented by any sudden depression of power, as for example by shock, by hæmorrhage. When the system is thus exhausted, convulsions are often abrupt in their appearance, and overwhelming in their effect, as in the cases last related. This occurs most frequently in infants, and is chiefly owing to the powerless and unresisting condition of the body; for the irritation acting singly, i. e. unaccompanied by such depression, gives a more permanent character to the convulsions.

"In short, there is an active and a passive form of convulsions, and to the proper treatment of the disease upon which they are symptomatic attendants, the understanding of this distinction is of the last importance. In one case we look for relief to cordials and tonics, in another to venesection and purgatives.

"In the commencement of inflammatory diseases, whether affecting the brain or other organs, convulsions, if present, are of the sthenic kind, and subside by the free use of the lancet; but if this treatment be pushed too far, or the inflammation should terminate in effusion or disorganization, the powers of life yield, and they become asthenic or passive, i. e. symptomatic of exhaustion. Such are the convulsions which appear after hemorrhage or any other rapid depression of the system, and in the last stage of acute diseases. The smallest direct reduction of strength will, in such cases, extinguish life. It can only be maintained by the timely administration of such aliment and medicine as support without exciting, and therefore tranquillize the system." 144.

It is with this form of convulsions that we have to do in most cases of prostration following injuries and operations; but not in all.

### INFLAMMATION SUCCEEDING INJURIES AND OPERATIONS.

Mr. Travers next proceeds to exemplify the phenomena of *direct irritation* arising from the inflammation that ensues on injuries and operations, by a detail of cases. Some of these we must necessarily analyze, in order to keep up the connexion of the subject.

*Case* I. A man, aged 40, was admitted into Guy's Hospital, January 1st, for a superficial collection of matter in the palm, with a severe inflammatory œdema of the whole hand and fore-arm. It had been caused by a bruise with a hook five days previously. The abscess was freely opened, and two ounces of sanious matter discharged. He was in a state of severe irritation, as indicated by his countenance and manner. His eyes looked glassy—pupils contracted—pulse small and quick—tongue much furred. In the night all these symptoms were aggravated—delirium came on, and next day (seventh from the injury) he died, the whole arm being in a state of gangrene.

*Case* 2. A woman, aged 66 years, pricked her thumb, while washing with pearl-ash, in November. The next day the finger became much swelled and painful, which, on the third, extended to the hand and wrist, the inflammation spreading alarmingly upon the fore-arm. The pain was excruciating, and the constitutional disorder extreme. The pulse was small, quick, and jerking—tongue dry and foul—oppression of the præcordia—total failure of appetite—thirst—great restlessness and anxiety.

"After repeated and copious leech-bleedings, continual fomentations, free purging with calomel, and the exhibition of antimonial opiates, the swelling and redness subsided, and the inflammation was arrested in its course, midway between the shoulder and elbow, on the eighth day from the accident. But the hand now assumed a livid cast; large phlyctenous vesications appeared, both on the palm and dorsum; the pulse was very small and feeble; the tongue covered with a dry and dark crust; she was exceedingly depressed, and refused both wine and nourishment. In the evening and during the following night, she was violently delirious, and with difficulty kept in bed; in the morning she appeared exhausted; stupor succeeded, and she died in that state on the ninth day from the injury, at one p. m. the hand and arm presenting a state of sphacelus." 148.

*Case* 3. Laurisson, aged 17, a healthy lad, received a severe wound from a piece of timber on the 29th November. On being received into Guy's Hospital, it was found that the integuments in front of the knee-joint were lacerated, so as to expose the insertions of the vasti and rectus muscles. There was also an aperture in the hollow of the ham, into which the little finger might be admitted. There was little hæmorrhage and no escape of synovia, so that the joint was considered safe. The edges of the wound were brought together, and the limb placed in a relaxed posture. In the evening he complained of cold and numbness in the injured limb—his whole system seemed much depressed—and he was very drowsy—pulse 120, and small. Camphor julep, liq. ammon. acetat. and laudanum, every four hours. Next day, much tension in the knee and calf of the leg—knee poulticed. Symptoms of irritation are increasing. Third morning, the constitution is strongly sympathising, and great general debility is present—leg cold, clammy, and senseless, on the verge of gangrene. On the 5th day, at noon, he died.

On examination, neither the popliteal artery nor nerve, nor any principal branch appeared to have been wounded. The popliteus muscle was torn across, and a small orifice was discovered in the capsule of the joint, behind the external lateral ligament. The synovial membrane lining the external half of the joint was highly inflamed. Much blood was extravasated among the muscles in the neighbourhood of the joint.

The following observations are so judicious and important that we shall give them in the words of the author.

"These cases exemplify the rapid and fatal termination of acute inflammation of the tela cellulosa, consequent upon slight wounds, in extraordinary constitutional irritation. This is by no means, however, a legitimate consequence of the mischief inflicted. When such cases have been permitted by the patient's neglect, or slight appreciation of an injury apparently trifling, to reach a certain point, the efforts of art are often ineffectual to preserve life. The patient in this class of life seldom applies for proper assistance until, to use his own phrase, he "feels ill all over." Then we find him labouring under the established symptoms of constitutional irritation. A contracted and quick pulse, foul and encrusted tongue, rigors and flushes, great anxiety, bewildered expression, constant vigilance, diffused pain, &c. Then follow increased rapidity and intermission of the pulse, cold clammy surface, hiccup, subsultus, muttering, or paroxysms of frenzy, stupor, and death.

"This is a description of case which happens yet more frequently in the lower limbs. An old leg ulcer—a slight recent injury, as an abraded instep, ancle, or shin—a diseased toe-nail, an inflamed corn, or ganglion, irritated to acute diffused inflammation of the cellular membrane of the limb, gives origin to a constitutional state over which medicine has little control. Unquestionably the susceptibility to such a state is greater in the aged, the dram-drinker, the man of broken constitution; but in these the aggravation is less because the constitution sooner takes alarm. The robust and healthy, relying on the soundness of constitution, quickly reach the same perilous crisis by braving the evil. 'This ought not to have been,' is the instant impression which the sight of the case conveys. This the patient always feels, and often expresses; but to look back is as little consolatory as the prospect. I have known eminent practitioners prescribe calomel and jalap every six hours, within two days of the patient's decease, in the belief that so vitiated a condition of the visceral secretions as of course and consequence exists, is yet the gravamen of the mischief. At the same time wine and strong nourishment have been proscribed. This is the ultraism of faith in certain doctrines unimpugnable when unabused, but capable, like every thing excellent, of being injured by a blind devotedness. The most important practical indications which these cases convey are, 1st. early and free venesection to the relief of pain; 2nd. early and free openings of abscesses. If these are overlooked, the effective aid of medicine is questionable; if they are fulfilled, it is capable of affording most essential benefit, both in the stage of excitement and collapse."—158.

The common error, Mr. Travers observes, is reliance on local blood-letting till general blood-letting is inadmissible.

"Under a prudent restriction, pain may be taken for a director to the use of the lancet, even in incipient gangrene." It is not the process of mortification, our author remarks, which destroys in these cases;—but the irritation of the nervous system by the inflammation, and the acutely-agonizing pain which accompanies it. When the powers of life are well nigh exhausted, the texture of the part becomes broken up by the disease, and the phenomena of gangrene are presented. This is an effect, not a cause, of the constitutional malady.

"The general impression which the appearance of gangrene on the eve of dissolution has given, is erroneous. The part is disorganized, and no longer retains the principle of resistance to decomposition, but the mischief is done before the discoloration and bloody vesicles appear; nay, it as often happens before the part has so lost its organization as to part with its vitality. How many instances do we see in which with very moderate constitutional disturbance, gangrene passes on to sphacelus, and limbs are separated, to use Mr. Hunter's phrase, by 'the natural surgeon.' But the power required for the process of separation depends upon a less disturbed state of the system, a state comparatively tranquil, and opposed, in all respects, to that of direct irritation." 159.

*Case* 4. Anne Pearson, aged 36, was admitted into St. Thomas's Hospital for a tumour on the inner side of the tibia, near its lower extremity. The enlargement of this tumour had been marked by lancinating pains, and it had a knotty and uneven surface, discoloured by a number of superficial vessels contorted and diffused thereon. There was no apparent distention of the larger veins. It was four inches in length and three in breadth. Pressure was tried, but without success, and on Friday, the 27th July, the tumour was cut out. So strong and intimate were its adhesions to the skin that it could scarcely be separated therefrom; while its attachments to the subjacent strong fascia near the bone, covering the muscles of the leg, was but slight, and easily broken through. The substance of the tumour was of a fatty nature, connected by numerous vessels. Next day, there was symptomatic fever, with an inflammatory redness extending up the leg and thigh, along the absorbents. Pulse 110. On the fourth day the inflammation had extended as high as the groin, and the inguinal glands were enlarged. No disposition in the wound to heal—no appetite—tongue much furred—leg and thigh leeched and fomented. Three grains of calomel and ten of rhubarb every six hours. *Fifth day*, The inflammation has subsided a good deal—less nausea and sickness—skin hot and dry—tongue cleaner. Four or five motions from the rhubarb and calomel. This state of amelioration continued for the succeeding day, but on the seventh day, she complained of violent pains in her right arm and down the right side and leg. A patch of redness appeared on the arm, which became diffused in the night. *Eighth day,* The redness changed to black, with delirium and incessant talking, great thirst, &c. She died in the night. The only morbid appearance that could be discovered, upon careful inspection of the body, was a chronic and firm adhesion between the costal and pulmonary pleuræ, in both sides of the chest.

*Case* 5. A young man, a baker, a sober healthy man, underwent amputation of the ring finger, at the metacarpal bone, in consequence of its having become stiff by a chronic inflammation from injury. The integuments were, at the time, in an unfavourable state—adhesion did not take place—and the wound turned sloughy. Some secondary hæmorrhage also took place. Although the loss of blood was not considerable, the man was evidently rendered irritable by it, and the appearance of the wound was altered for the worse. No serious constitutional disturbance, however, occurred till the eleventh day, when he experienced a severe rigor, and then became feverish and irritable. Calomel and antimony every six hours. From this time the symptoms gradually got worse, and he died on the 16th day from the operation.

This and a few other events of a similar kind are calculated to excite alarm in the mind of the surgeon, when about to remove a sound finger or toe. Sir Astley Cooper, however, whose experience is so extensive, informs us that he entertains no apprehension on this point, as he has met with no unfavourable termination of this description for many years. Immediately succeeding the case above detailed by Mr. Travers, is another, where a young man had a toe removed in consequence of a caries, with a fistulous opening, which had resisted every means of treatment. The operation was followed by pain in the wound and a restless night. Some unpleasant symptoms which followed were relieved, and he became convalescent ; but cough came on, the appetite failed, disturbance of the mental functions succeeded, and he died hectic, within a month from the operation. On examination, extensive adhesions were found on both sides of the chest—the substance of the lungs sound—some effusion between the membranes of the brain—and a large collection of pus was discovered beneath the flexor tendons in the sole of the foot, a circumstance that was not at all suspected during life.

### HÆMORRHAGE AND COLLIQUATIVE SUPPURATION.

The state of direct irritation is often produced, as every practitioner knows, by exhaustion from *loss of blood*, concomitant with, or consequent on, injury—and also by what our author calls colliquative suppuration.

"A hæmorrhage which does not prove directly fatal, as from a wounded artery, sometimes leaves the patient in a state of exhaustion so great that he is incapable of sustaining the shock of an operation. In lacerated, especially gun-shot wounds, complicated with fracture, a person is occasionally reduced to such extremity of weakness by loss of blood, that it becomes a question very difficult to decide, whether he will survive the removal of the injured part, if that measure be necessary." 183.

A man was sent to Mr. Travers from the country, a distance of ten miles, with a shattered arm, occasioned by the bursting of a gun. A tourniquet had been placed on the limb, which the man's wife had, by mistake, relaxed on the road, and thereby produced a large flow of blood. He was brought to the hospital in a state of exhaustion. Two hours were suffered to elapse before the operation (which was indispensable) in order to ascertain his condition. His pulse was now 60 in the minute, neither thready nor intermitting—faculties clear—limbs warm—complexion improved. In the operation (above the elbow) scarcely an ounce of blood was lost, and he preserved great equanimity of mind till the limb was removed, when he fainted, but soon recovered. Although closely watched, and well plied with cordials, the faintings returned at intervals, and he expired on the evening of the following day.

A great loss of blood during or subsequent to an operation, is often attended with serious consequences of another kind. It so prostrates the vital powers as to expose the parts to imminent hazard of erysipelas and gangrene.

"I have known a case of the excision of hemorrhoids, from which a considerable quantity of blood had been for some time previous discharged at the daily stool, terminate in erysipelatous inflammation of the mucous membrane of the rectum, which in a few days destroyed the patient. A similar result where the patient has been much debilitated, has been known to follow the operation of tying a portion of the gut in prolapsus ani; and in one remarkable instance a copious secretion of pus was found upon the interior membrane of the bowel, and also in the hemorrhoidal veins; but this patient was, at the time of the operation, the subject of scrofulous tubercles in the liver.

"When the system has been rendered irritable, but is recovering, either from hemorrhage, or from an injury or inflammation unattended by hemorrhage, a secondary bleeding, even though it be inconsiderable, often extinguishes life. This is the case in wounded arteries which have been unadvisedly trusted to the compress and bandage; and in deep suppurations, where a vein is opened by ulceration. I have seen persons really recovering from severe injuries, thus suddenly carried off. The symptoms in these cases are those of sinking, or pure prostration.

"The loss of blood by injury, when insufficient to destroy life by syncope, induces that state of the arterial system which prevails in gangrene; in other words it converts healthy inflammatory, or sympathetic fever into the asthenic excitement which accompanies prostration: and the inflammation of healing (granulation) into that of destruction (sloughing). Debility is the basis of morbid irritation, and those causes of debility which operate with the greatest force and directness, most invariably aggravate the state of irritation.

"These I call examples of death by irritation assuming the character of prostration, because, however the cause by which direct debility is induced may differ from that which operates in shock, where little or no blood escapes, the symptoms bear a close resemblance, and are equally to be referred to a great and sudden reduction of power with a local irritant. The loss of blood is not fatal in cases in which the circulation, although feeble in the extreme, recovers and maintains its regularity, and no excitement remains, as in uterine flooding after delivery; but where an extra burthen lies, or is imposed, upon the system, whether the unrelieved uterus, the unrecovered shock, the mutilated limb, or the removal of it, the vital powers succumb. It is very questionable whether the administration of stimulants is not more frequently injurious in these circumstances than beneficial. Excitement to increased action where the power is so much reduced as to be scarce able to maintain that which is necessary to life, only the more rapidly exhausts it. Hence it has been a good turn of fortune for many persons to have been left for dead, as it is called, on the field of battle. Sleep will restore where alcohol destroys. And whether life thus reduced in power can be maintained artificially in any case, i. e. where the patient being left to himself, the relief of natural sleep comes not to his aid, or comes in such a shape as not to refresh, may well be doubted. I am sure I have seen cases in which the remnant has been more quickly consumed by the incessant appliance of stimulus, than would have been the case had nature been left to her own economy. On the other hand, it must be admitted, in cases of such extreme prostration as is indicated by the entire relaxation of the sphincters, that the very sparing, but frequent supply of a nutrient liquid, a tea-spoonful at a time, or of a stimulus so diluted as not sensibly to swell the pulse, has sometimes succeeded in preserving the life 'of him that was ready to perish.'" 188.

*Excessive Suppuration.* This will so sink the vital powers as to induce a state of direct constitutional irritation in an extreme degree. Three cases are introduced by Mr. Travers in exemplification of the effects of colliquative suppuration. Of these we do not deem it necessary to quote more than one, being a rare case of profuse and exhausting suppuration, directly supervening upon the amputation of a crushed limb, where the prostration obviously occasioned by it, and progressively increasing, terminated in the death of the patient on the eleventh day.

*Case.* "Izzard, a drayman, aged forty-two, fell from the shaft of his dray, and one wheel passing over his right leg produced a comminuted fracture, with incurable laceration of the soft parts. The injury was attended by a free hemorrhage. Amputation was performed immediately above the knee; no symptom of extraordinary irritation ensued, but a considerable swelling of the thigh was noticed two days after the amputation. On the third it was opened, and a profuse and fetid discharge issued from it. On the fifth day, the swelling had so much increased as to make the thigh appear half as large again as the other. The surface was tense, and covered with a blush of inflammation. The integument on the face of the stump, which was abundant, was loose, flaccid, and discolored; no adhesive process had been set up; the divided muscles had a ragged and sloughy appearance; the discharge was thin and of a bad color, and excessive in quantity. The man made little or no complaint, but his pulse was very quick, weak, and compressible. Warm spirituous fomentations were applied to the limb; he was ordered bark and opium, and a quart of porter per diem. His bowels had been kept regular from the first with Epsom salts in mint julep occasionally. From this time to his dissolution the man's symptoms were simply those of great and daily increasing debility. He passed much of his time in sleep, which was disturbed by starting and was unrefreshing. The discharge continued profuse, and of nearly the same quality, although the blush upon the skin had disappeared, and the size and tension of the limb were much reduced, so that the skin had collapsed and the swelling did not extend to the groin. He always drank his porter with relish, and even asked for rabbit for his dinner the day before his death. This event, which was preceded by the ordinary signs of exhaustion, took place on the eleventh day from the accident." 198.

We have now brought our analysis forward to the important subject of POISONS—chiefly animal—or in other words, the effects of *dissection wounds*, which subject occupies nearly two hundred pages of the volume, and will supply ample materials for our next article. In a third, we hope to complete our view of this important work, which becomes much more interesting as we proceed. It will be seen that, as far as we have yet gone, the work consists principally of cases in illustration—and of these we have endeavoured to present our readers with a very full view. It will probably have occurred to the reader, that the arrangement which Mr. Travers has adopted, has not been very conducive to that "lucidus ordo" which is the object of all arrangements. But we are ready to grant that it would have been exceedingly difficult, on any plan, to avoid the appearance of tautology in the elucidation of irritation as arising from various sources, and in various ways from the same source. On this account we are not inclined to cavil at trifles, when so much important matter is brought before us from the most authentic records—those of a public hospital. But the time for general comments will be at the close of our analysis; and till then we part from our able and intelligent author with feelings of great respect and esteem.

---

